# LANA Binds to Multiple Active Viral and Cellular Promoters and Associates with the H3K4Methyltransferase hSET1 Complex

**DOI:** 10.1371/journal.ppat.1004240

**Published:** 2014-07-17

**Authors:** Jianhong Hu, Yajie Yang, Peter C. Turner, Vaibhav Jain, Lauren M. McIntyre, Rolf Renne

**Affiliations:** 1 Department of Molecular Genetics and Microbiology, University of Florida, Gainesville, Florida, United States of America; 2 UF Genetics Institute, University of Florida, Gainesville, Florida, United States of America; 3 UF Health Cancer Center, University of Florida, Gainesville, Florida, United States of America; University of North Carolina at Chapel Hill, United States of America

## Abstract

Kaposi's sarcoma-associated herpesvirus (KSHV) is a γ-herpesvirus associated with KS and two lymphoproliferative diseases. Recent studies characterized epigenetic modification of KSHV episomes during latency and determined that latency-associated genes are associated with H3K4me3 while most lytic genes are associated with the silencing mark H3K27me3. Since the latency-associated nuclear antigen (LANA) (i) is expressed very early after *de novo* infection, (ii) interacts with transcriptional regulators and chromatin remodelers, and (iii) regulates the LANA and RTA promoters, we hypothesized that LANA may contribute to the establishment of latency through epigenetic control. We performed a detailed ChIP-seq analysis in cells of lymphoid and endothelial origin and compared H3K4me3, H3K27me3, polII, and LANA occupancy. On viral episomes LANA binding was detected at numerous lytic and latent promoters, which were transactivated by LANA using reporter assays. LANA binding was highly enriched at H3K4me3 peaks and this co-occupancy was also detected on many host gene promoters. Bioinformatic analysis of enriched LANA binding sites in combination with biochemical binding studies revealed three distinct binding patterns. A small subset of LANA binding sites showed sequence homology to the characterized LBS1/2 sequence in the viral terminal repeat. A large number of sites contained a novel LANA binding motif (TCCAT)_3_ which was confirmed by gel shift analysis. Third, some viral and cellular promoters did not contain LANA binding sites and are likely enriched through protein/protein interaction. LANA was associated with H3K4me3 marks and in PEL cells 86% of all LANA bound promoters were transcriptionally active, leading to the hypothesis that LANA interacts with the machinery that methylates H3K4. Co-immunoprecipitation demonstrated LANA association with endogenous hSET1 complexes in both lymphoid and endothelial cells suggesting that LANA may contribute to the epigenetic profile of KSHV episomes.

## Author Summary

KSHV is a DNA tumor virus which is associated with Kaposi's sarcoma and some lymphoproliferative diseases. During latent infection, the viral genome persists as circular extrachromosomal DNA in the nucleus and expresses a very limited number of viral proteins, including LANA, a multi-functional protein. KSHV viral episomes, like host genomic DNA, are subject to chromatin formation and histone modifications which contribute to tightly controlled gene expression during latency. We determined where LANA binds on the KSHV and human genomes, and mapped activating and repressing histone marks and RNA polymerase II binding. We found that LANA bound near transcription start sites, and binding correlated with the transcription active mark H3K4me3, but not silencing mark H3K27me3. Binding sites for transcription factors including znf143, CTCF, and Stat1 are enriched at regions where LANA is bound. We identified some LANA binding sites near human gene promoters that resembled KSHV sequences known to bind LANA. We also found a novel motif that occurs frequently in the human genome and that binds LANA directly despite being different from known LANA-binding sequences. Furthermore, we demonstrate that LANA associates with the H3K4 methyltransferase hSET1 which creates activating histone marks.

## Introduction

Eukaryotic DNA is packaged into chromatin which plays a central role in the regulation of all DNA processes including replication, transcription, and repair. Chromatin contains nucleosomes with DNA wrapped around the core histones H2A, H2B, H3, and H4. Nucleosomes carry epigenetic information in the form of post-translational histone modifications. N-terminal histone modifications including acetylation, methylation, phosphorylation, and sumoylation are important in partitioning chromatin into transcriptionally active or repressive domains (reviewed in [1]). In mammalian cells, genome-wide ChIP-seq assays revealed that histone acetylation at H3K9 and H3K4 trimethylation (H3K4me3) correlate with active transcription, while H3K27 trimethylation (H3K27me3) is detected in promoters of repressed genes [2]. The apparently opposite modifications H3K4me3 and H3K27me3 co-localize at some promoters (“bivalent marks”), poising these genes to be transcribed upon signaling. Histone modifications are also detected in regions outside promoters. All three states of H3K4 methylation are highly enriched at insulator sites, while only H3K4me and H3K4me3 are associated with enhancers [2], [3].

Histone lysine methylation is mediated in mammalian cells by a large family of lysine methyltransferases (KMTs) that exist in protein complexes. A single enzyme can be responsible for the three states of methylation in a progressive manner, or different enzymes may be required for different methylation states. Mammalian cells contain 10 different H3K4 KMTs, which include the hSET complex, mixed lineage leukemia 1 to 5 (MLL1-5) complexes, Set7/9, Smyd1, Smyd3, and Prdm9, which are largely not redundant [3], [4], [5]. hSET1 and MLL complexes share three core components: WDR5, RbBP5, and ASH2L, and siRNA-mediated knockdown of these proteins leads to a significant reduction of global H3K4 methylation, strongly suggesting that hSET1 and MLL are responsible for the majority of H3K4 methylation [6], [7]. It has been demonstrated that the hSET1 and MLL complexes can be recruited to specific promoters through interactions with transcription factors or co-activators including E2F, NF-E2, MAPK, and USF1 [8], [9], [10], [11]. Moreover, for HSV-1, an α-herpesvirus, it was demonstrated that hSET1 or MLL complexes are recruited to IE promoters through a VP16/HCF interaction, the latter functioning as a scaffold for the activator complex [12], [13].

Kaposi's sarcoma-associated herpesvirus (KSHV, also named HHV8) is a γ is a named. In addition to Kaposi's sarcoma (KS) which targets endothelial cells, KSHV is associated with two lymphoproliferative disorders: primary effusion lymphoma (PEL) and a subset of multicentric Castleman's disease (MCD). Although the majority of cells in KS tumors are latently infected, both latent and lytic phases of KSHV infection contribute to pathogenesis and tumorigenesis [14]. During latency, viral gene expression is restricted to a small subset of genes including the latency-associated nuclear antigen (LANA), vCyclin, vFLIP, Kaposins, and viral miRNAs [15], [16].

LANA is a multifunctional protein with important roles during viral latency. LANA is the only viral protein required for episome maintenance by supporting KSHV latent DNA replication and tethering the viral episome to cellular chromosomes. Tethering is mediated by LANA via interactions with the viral terminal repeats and the core histones H2A/H2B [17], [18], [19], [20], [21]. LANA associates with several chromatin modifying complexes including the histone H3 lysine 9 (H3K9) methyltransferase SUV39H and the H3K9 demethylase KDM3A [22], [23]. In addition, LANA binds to the histone acetyltransferase CBP and histone deacetylase complex mSin3 [24], [25]. LANA contributes to activation and repression of host and viral genes, presumably by interacting with transcriptional activators (i.e. Brd2/4 or RING3, Sp1, Ap1) [26], [27], [28], [29] and repressors (HP1, Dnmt3, and mSin3) [24], [30], [31] and proteins involved in chromatin remodeling (FACT and CBP) ([25], [32]; for recent review see [33]).

We hypothesized that LANA plays a role in the establishment and maintenance of the KSHV epigenome. To address this question and to identify viral and cellular genes potentially regulated by LANA, we performed genome-wide ChIP-seq analyses for LANA, Pol II, and histone modifications. Our data confirm that during latency both active H3K4me3 and repressive H3K27me3 marks are associated with the viral episomes [34], [35]. Interestingly, H3K4me3 marks are highly correlated with LANA occupancy at sites where the silencing mark H3K27me3 is excluded. Furthermore, it was demonstrated that LANA selectively associates with H3K4 lysine methyltransferase (KMT) hSET1 complexes. Our data suggest that LANA may directly contribute to the viral epigenome by binding to specific viral promoters and enhancers and by interacting with H3K4 KMT hSET1 complexes.

## Results

### Genome-wide occupancy of H3K4me3, H3K27me3, and RNA Polymerase II on KSHV episomes in BCBL-1 cells

Histone marks on KSHV genomes have previously been mapped by PCR-based ChIP assays and ChIP-on-chip assays using tiling array hybridization [34], [35], [36], [37]. We investigated the KSHV epigenome using ChIP-seq, which has been applied to the genome-wide analysis of epigenetic modifications in mammalian cells [1], [2].

Based on studies demonstrating that the patterns for acetylated H3K9 and H3K14 were almost identical with H3K4me3 on the KSHV epigenome [34], [35], we characterized the transcription-associated mark H3K4me3 and the repressive mark H3K27me3 in combination with RNA polymerase II (Pol II). ChIP-seq assays were performed in BCBL-1 cells with antibodies against H3K4me3, H3K27me3, and Pol II. Sequencing reads were sequentially aligned against KSHV (accession number NC_009333) and human genome hg19 using Bowtie [38]. About 89.8–95.9% of tags were aligned to hg19 and 0.73–2.4% to KSHV. To determine reproducibility of ChIP-seq assays, we compared two biological replicate datasets of H3K4me3 ChIP-seq in BCBL-1 cells using a Bland-Altman analysis [39], [40], [41], [42]. As shown in Figure S1, the 95% confidence interval shown between the green lines indicates high reproducibility. Data have been submitted to NCBI GEO (accession number GSE52421).

**Figure 1:**
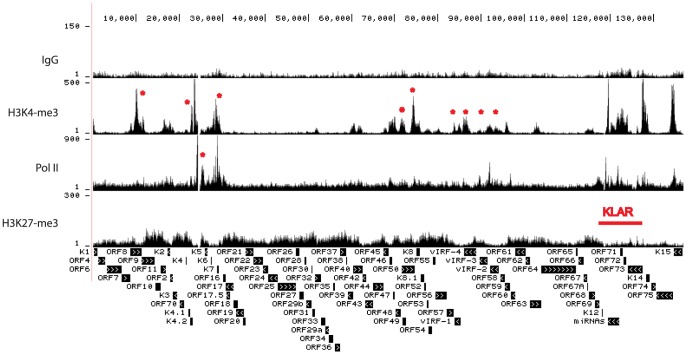
Genome-wide epigenome analysis of KSHV in BCBL-1 cells. ChIP assays were performed in BCBL-1 cells with control IgG, H3K4me3, H3K27me3, and Pol II antibody. The resulting ChIP DNA was used for library construction followed by Illumina sequencing. Sequencing tags mapped to viral genome were visualized in UCSC Genome Browser. The number of sequence tags is indicated for each track (y-axis), which represents the number of times each region was recovered by the ChIP-seq. The highest coverage for H3K4me3 is 6,660 within the terminal repeat which was not shown in order to visualize the lower peaks.

ChIP-seq tags mapped to KSHV were used for peak analysis by CisGenome [43]. Genome-wide profiling of H3K4me3, H3K27me3, and Pol II occupancy on the KSHV genome in BCBL-1 cells was visualized using the UCSC Genome Browser (Fig. 1). Previously published nucleotide (nt) numbers from Gene Bank accession # U75698 are converted to accession # NC_009333 as referred to in Table S1. Control IgG gave low background while H3K4me3 and H3K27me3 yielded specific occupancy patterns (Fig. 1). Within the unique long region, multiple H3K4me3 peaks are located at the KSHV latency-associated region (KLAR) including the LANA (ORF73) promoter, a broad region from the beginning of ORF72 coding sequence to the beginning of the miRNA cluster, and the intragenic region in between K12 and miRNAs (Fig. 1). The latency-associated region is a complex locus containing at least three promoters driving the expression of LANA, vCyclin, vFLIP, miRNAs, and the Kaposin family of proteins [26], [44], [45]. Distribution of H3K4me3 in this region is shown in more detail in Fig. S2, and is consistent with expression of this region during latency. In addition, several lytic genes including ORF8/ORF9, K4.2, ORF50 (RTA), K7, K8, vIRFs and ORF58 were enriched for H3K4me3 at different levels (Fig. 1, marked by asterisks). Unlike H3K4me3 which forms distinct peaks, H3K27me3 is distributed more broadly across large regions containing late lytic genes that are void of H3K4me3 (Fig. 1). Pol II occupancy was probed with an antibody that recognizes both elongating and pausing Pol II [46], [47], [48] and displayed a number of distinct peaks within the latency-associated region that coincide with H3K4me3. Outside of this region, the highest Pol II occupancy was detected in a region spanning ORFs K4.2 to K7 (asterisk). At most genomic loci H3K4me3 and H3K27me3 are mutually exclusive. Several blocks of lytic late genes, including loci from the beginning of the genome to 9.5 K, regions spanning 30 K to 60 K and 77 K to 83 K, are enriched with H3K27me3 but void of H3K4me3 and Pol II, indicating heterochromatin structure (Fig. 1).

**Figure 2:**
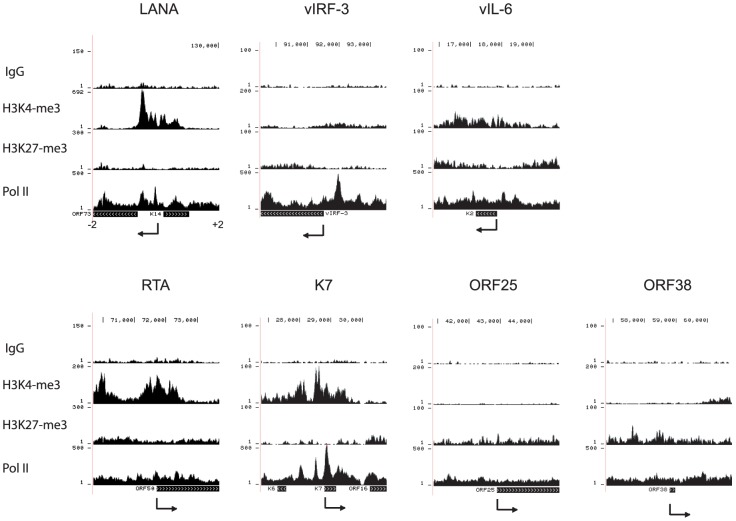
H3K4me3 and H3K27me3 modifications and PolII occupancy at selected viral promoters in BCBL-1 cells. The sequencing tags were mapped to the viral genome. The +2 to −2 Kb regions spanning the transcription start site were screened for the occupancy by H3K4me3, H3K27me3, and Pol II. The arrows indicate the transcription start site and orientation of transcription.

We focused in more detail on H3K4me3, H3K27me3, and Pol II occupancy at the +/−2kb region surrounding known transcription start sites (TSS) of selected viral genes (Fig. 2). Promoters for the latent genes ORF73 (LANA) (Fig. 2) and vIRF-3 are enriched for H3K4me3 and Pol II but depleted for H3K27me3, as was the transcription start site for vIL-6 (Fig. 2). Recent transcriptome profiling and chromatin structure analysis showed that the vIL-6 promoter is active in a subpopulation of PEL cells during latency [49], [50]. Promoters for many additional viral genes displayed enrichment for H3K4me3 and Pol II. For example, the promoter of the lytic gene K7 is significantly enriched with H3K4me3 and Pol II, but depleted for H3K27me3. Although these epigenetic marks suggest transcriptional activity, it was recently demonstrated by Toth et al. that transcription of K7 is paused at the elongation step through NELF binding to pol II [51]. As expected, lytic late gene promoters are enriched for H3K27me3 as exemplified by ORF25 and ORF38, encoding a major capsid protein and a tegument protein, respectively (Fig. 2). The promoter for the lytic immediate early gene RTA is enriched for Pol II and both H3K4me3 and H3K27me3 (bivalent marks, Fig. 2).

**Figure 3:**
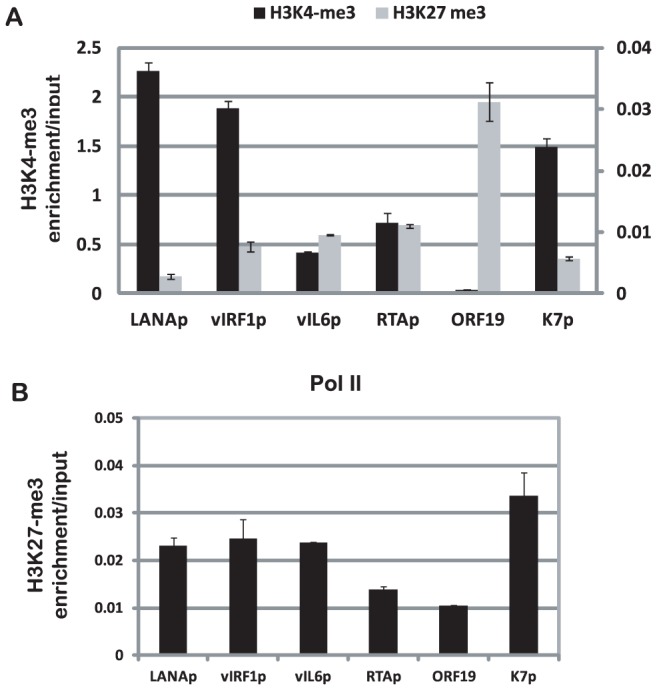
Validation of ChIP-seq data by quantitative PCR. Quantitative PCR assays were performed with ChIP DNA to detect the enrichments of H3K4me3 and H3K27me3 (A), and Pol II (B) on promoters for LANA, vIRF1, vIL6, RTA, coding region of ORF19, and K7. The level of enrichment of chromatin immunoprecipitated by antibodies was normalized to input.

For validation, quantitative ChIP-PCR assays confirmed ChIP-seq results for the promoter regions of LANA, vIRF1, vIL6, RTA, and K7, and the coding region of the late lytic gene ORF19, which was enriched with H3K27me3 (Fig. 3). In summary, these H3K4me3 and H3K27me3 ChIP-seq profiles are in agreement with the general patterns from previously published ChIP-on-chip studies [34], [35].

### Epigenome of KSHV in long-term infected TIVE cells

**Figure 4:**
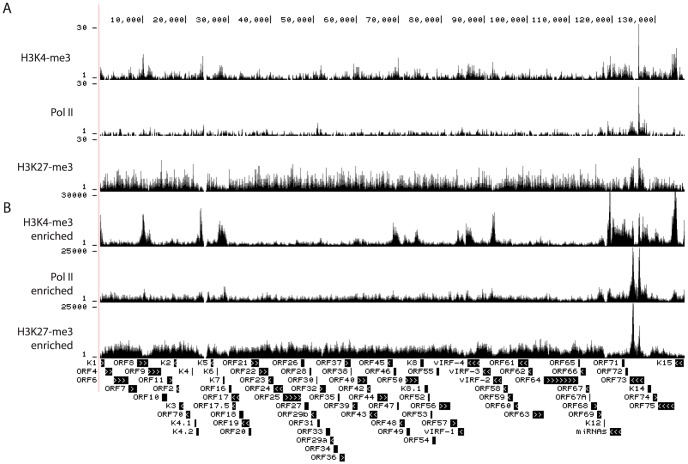
Occupancy by H3K4me3, H3K27me3, and Pol II on the viral episomes in TIVE-LTC cells. ChIP-seq was performed in long-term infected TIVE cells with antibody against H3K4me3, H3K27me3, or Pol II. The corresponding library was sequenced without (A) or with (B) enrichment for viral sequences with SureSelect Target Enrichment System (details in Material and Methods). Tags were mapped to viral genome and visualized in UCSC Genome Browser. The number of sequence tags is indicated for each track (y-axis).

To date, epigenetic modifications have only been mapped in cells of lymphoid and epithelial cells but not in endothelial cells, which give rise to KS. To address this gap in the literature, we performed ChIP-seq assays in long-term infected TIVE-LTC cells, which contain BCBL-1 derived episomes [52], [53]. Genome-wide occupancy of H3K4me3, H3K27me3, and Pol II is depicted in Figure 4A. The viral copy number in TIVE-LTC is less than 5 copies per cell, which is comparable with KS lesions *in vivo* [53]. As a result, the total number of KSHV-specific sequence tags was 25- to 165-fold lower in TIVE-LTC compared to BCBL-1 (Compare y-axis in Fig. 4A and Fig. 1). Because the total number of reads for KSHV in TIVE-LTC cells was low, we increased coverage by applying SureSelect target enrichment technology (Agilent). ChIP-seq libraries were incubated with a custom-designed KSHV-specific biotin-labeled RNA bait library which yielded 2,000- to 4,000-fold enrichment. While numbers of tags per peak increased, ChIP-seq profiles were similar to those observed without enrichment indicating non-biased selection (comparing corresponding tracks in Fig. 4A to Fig. 4B).

H3K4me3 occupancy showed significant differences in TIVE cells compared to BCBL-1 cells within the latency-associated region. A prominent H3K4me3 peak within the ORF73 (LANA) promoter (around nt 127,600) observed in BCBL-1, was demonstrably reduced in TIVE-LTC cells. Instead, a strong H3K4me3 peak appeared at nt 126,280 within the LANA coding region, which was not present in BCBL-1 cells (Fig. S2 panel B). Interestingly, in addition to H3K4me3 and Pol II, this region was also enriched for H3K27me3 thereby creating a bivalent mark. Outside of the latency-associated region occupancy of H3K4me3 was decreased at several areas including the K4.2 promoter region (around 23 K), ORF50 promoter region (around 72 K), 96 K, and 103 K region (Fig. 4). Conversely, H3K27me3 signals increased significantly within the LANA coding region (shown as two peaks covering nt 124.1 K to nt 125.5 K and nt 126 K to nt 127 K). Pol II occupancy was significantly decreased throughout, especially at the K4.2 promoter region (at about nt 24 K). In this context, it is interesting to note that TIVE-LTC cells are tightly latent [53] and we recently demonstrated that a subpopulation of episomes is heterochromatinized at the latency-associated region [49]. Whether the observed increased H3K27me3 and decreased H3K4me3 deposition are causative for the lack of reactivation in TIVE-LTC needs to be further investigated. In summary, except for a few changes affecting H3K4me3 deposition overall histone modification patterns were similar between PEL and endothelial cells.

### LANA occupies latent and lytic promoters during latency

In addition to its role in latent DNA replication and episomal maintenance, LANA is a key regulator of host and viral gene expression. LANA binds to DNA directly in a site-specific manner, or indirectly through protein-protein interactions with multiple chromatin associated proteins including core Histone H2A and H2B, CREB2, mSin3, RING3, MeCP2, SSRP1, and P53 (reviewed in [54], [55]). Hence, we hypothesized that LANA plays a role in the establishment and maintenance of the KSHV epigenome. To determine LANA occupancy on the viral and host genomes and to identify genes potentially regulated by LANA, we performed ChIP-seq using a monoclonal rat LANA antibody. Sequencing generated 5 million tags for rat IgG control, and between 14.7 and 36.7 million tags for BCBL-1, and TIVE-LTC cells with and without target enrichment.

**Figure 5:**
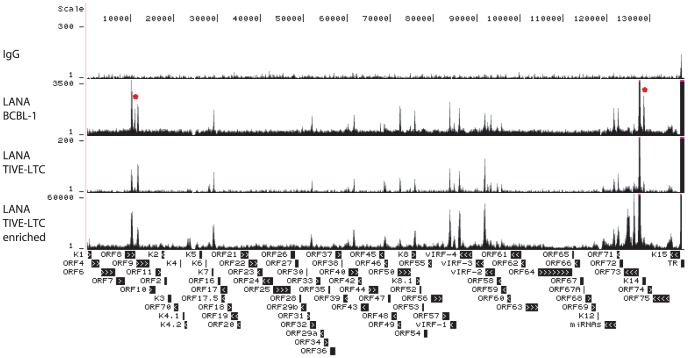
UCSC Genome Browser view of LANA binding on the KSHV genome. ChIP-seq was performed in BCBL-1, TIVE-LTC with or without SureSelect Target Enrichment System with LANA-specific antibody. Sequencing tags were mapped to viral genome and visualized in UCSC Genome Browser.

Genome-wide binding of LANA is depicted in Figure 5 and major peaks observed in BCBL-1 are listed in Table 1. Three LANA binding sites (LBSs) have previously been characterized by EMSA *in vitro*; two are located within the TR and contribute to latent DNA replication [20], [21], [56] and one is upstream of the LANA promoter, which is auto-regulated [24], [26], [44], [57].

**Table 1:** LANA binding peaks on KSHV genome in BCBL-1 cells.

Rank	Region	aStart (nt)	End (nt)	Average coverage
1	TR	137112	137336	1132065
2	TR	137810	138021	550626
3	ORF73 promoter	127391	127833	63511
4	ORF8	10146	10463	23610
5	K14	128517	128771	20716
6	miRNA cluster	121628	121849	19033
7	ORF8/ORF9	11636	11860	16718
8	vIRF1/vIRF4	85928	86151	16296
9	ORF57/vIRF1	83697	83915	15810
10	ORF71	122600	122813	15241
11	K7/ORF16	29147	29335	14573
12	ORF8/9	10896	11100	14563
13	vIRF2/vIRF3	91787	91991	14156
14	ORF50/	72220	72436	13357
15	K8	75623	75817	13328
16	ORF40/42	61602	61786	13266
17	ORF32/ORF33	51859	52018	12848

a nt coordinates are in accordance with accession number NC_009333.

LANA ChIP-seq revealed at least 17 distinct LANA peaks in both BCBL-1 and TIVE-LTCs and observed occupancy patterns are almost identical between both cell types (Fig. 5). However, two LANA peaks (marked by asterisks in Fig. 5) were clearly reduced in TIVE-LTC; one at nt position 11,636 within ORF 8 (Table 1 peak number 7) and one within the coding region of K14 at nt position 128,517 (Table 1 peak number 5). The two highest LANA peaks are within the TRs as expected. In agreement with *in vitro* data [20], LBS1 and LBS2 are strongly bound by LANA (Fig. S3). Consistent with a previous report, LANA also binds a region at the beginning of the TR, which is likely indirect since LANA failed to bind this site *in vitro* [58]. We observed strong LANA binding within the LANA promoter region; however, this LANA peak (nt 127,391 to nt 127,833) is located just downstream of the LANAp TSS at nts position 128,029 (Fig. S2B). Interestingly, the *in vitro*-characterized Sp1-containing LANA binding site (nts 128,051–128,072 upstream of the LANAp TSS) [26], [59], was not bound by LANA *in vivo*. Furthermore, this LANA peak completely overlaps with three CTCF binding sites (nt 127 514–127 693) [36], [60], [61], suggesting co-occupancy of LANA and CTCF, which was also observed at many host cellular promoters (discussed below). Three additional LANA peaks were detected within the latency-associated region located within the miRNA cluster, the ORF71 coding region, and the K14 ORF (Table 1 peak 6, 10, and 5 respectively). Several LANA peaks outside of the TRs and the latency-associated region (peak # 8, 9, 13, and 15) were located within a region previously reported to be bound by LANA *in vitro* [19]. The fact that none of these sequences showed sequence homology to LBS1/2 indicates that LANA binds either indirectly through protein-protein interactions or directly to sites with novel sequence-specificity.

**Figure 6:**
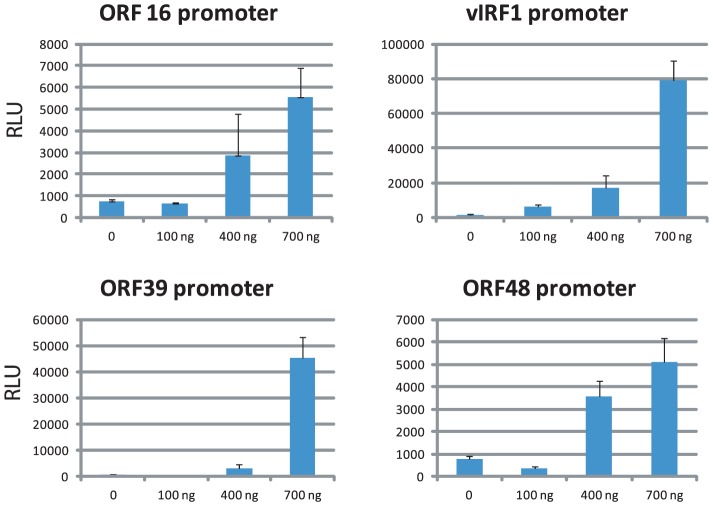
LANA transactivates viral promoters in a dose-dependent manner. The −2 Kb promoter regions of individual viral genes were PCR amplified and cloned upstream of Luciferase reporter gene. 293 cells were co-transfected with fixed amount of reporter plasmid and increasing amount of LANA expression vector as indicated. Results from three independent experiments are shown and error bars indicate standard deviations.

LANA negatively regulates RTA expression and it was demonstrated that the RBP-Jk sites within the RTA promoter are critical for LANA-dependent regulation [62], [63]. LANA binding was observed within the ORF50 (RTA) region; however, this LANA peak was not upstream of the TSS close to the RBP-Jk sites but instead 600 bp downstream within the ORF50 intron (Figure S3). Rosetto et al. reported LANA binding to oriLyt and modulation of viral lytic replication using *in vitro* replication assays [64]. However, LANA ChIP-seq did not reveal any LANA binding to oriLyt-L or oriLyt-R in BCBL-1 cells, which display a base level of spontaneous lytic replication. These data demonstrate that LANA binding to chromatin within the LANA and RTA promoters significantly differs from *in vitro* EMSA assays. Unexpectedly, numerous LANA peaks are located within promoters of IE, E, and late genes, including ORF16, ORF33, ORF39, ORF48, ORF58, ORF64, and vIRF-1 and -3 (Table 1). To determine whether LANA potentially contributes to their regulation, fragments 2 Kb upstream of their TSSs were inserted into luciferase reporter vectors and co-transfected with a LANA expression vector into HEK293 cells. As shown in Fig. 6, LANA transactivates the promoters of ORF16 (E), vIRF1 (E), ORF39 (L), and ORF48 (IE) in a dose-dependent manner, suggesting that LANA may contribute to lytic gene expression. Interestingly, in this context Wilson et al. identified a second LANA promoter (LANA_LTI_), which is RTA-responsive and induced during lytic replication [45]; however to date no functional role for LANA during lytic replication has been established. Hence, our transactivation data and observed LANA ChIP-seq profiles suggest that LANA binding during latency potentially affects viral genes of different kinetic classes.

### Identification of cellular genes bound by LANA in BCBL-1 cells and TIVE-LTC cells

**Figure 7:**
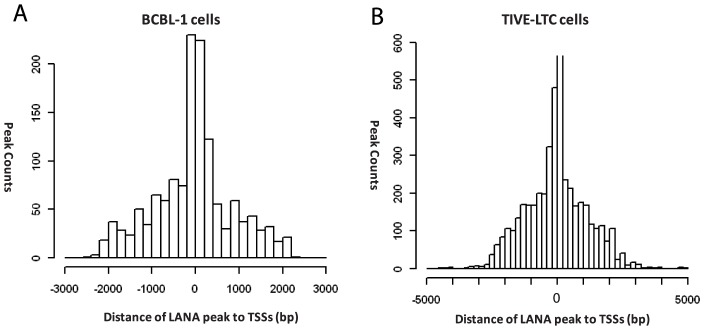
Positions of LANA peaks to the transcription start sites of known genes in BCBL-1 and TIVE-LTC cells. LANA peaks within the +/−2 kb region of known genes are calculated and plotted for distance from center of the peaks to the transcription start sites in BCBL-1 cells (A) and TIVE-LTC cells (B).

After viral mapping, unmapped reads from LANA ChIP-seq were mapped to the human genome hg19, and 2180 and 2951 unique peaks were identified in BCBL-1 and TIVE-LTC, respectively. In agreement with immunofluorescence data on LANA binding to mitotic chromosomes [17], [65], we additionally observed a large number of reads that aligned to highly repetitive GC-rich centromere regions. To focus on potential transcriptional targets, we identified LANA peaks within +/−2 kb relative to known TSS, which revealed a strong enrichment for LANA peaks around +/−500 bp in both cell types (Fig. 7). We identified 412 and 998 peaks located at promoter/enhancer regions upstream of 1295 (BCBL-1) and 3917 (LTC-TIVE) annotated transcripts, representing 167 and 505 identified gene symbols (Table 2, Fig. S5). Hence, LANA was detected at many more promoters in TIVE cells compared to BCBL-1.

**Table 2:** LANA occupancy on the human genome in BCBL-1 cells and TIVE-LTC cells.^a^

Cell type	# of Peaks called	Peaks in *promoter*			Peaks with similar LBSs
		Peaks	Transcripts	Gene Symbols	Peaks
**BCBL1**	2180	412	1295	167	58
**TIVE-LTC**	2951	998	3917	505	205

a Supplemental Figure S5 gives a flow chart representation of LANA ChIP peak analysis.

A list of all gene loci enriched by LANA ChIP-Seq in BCBL-1 cells and TIVE-LTC cells is provided in the supplement (Tables S2 and S3). While the observed LANA binding profile on the KSHV genome was nearly identical in lymphoid and endothelial cells, LANA binding to host genes is mostly cell type specific. Only 26 genes were commonly enriched between BCBL-1 cells and TIVE-LTC cells. While PARL, NIPAL2, IQGAP3 are BCBL-1 specific, MRPL53, NFYC, CCDC90B, and HIST2HBE were TIVE-LTC specific, while WDR74 showed nearly identical binding profiles in both cell types (Fig. S4). Two genes, BIRC6 (Survivin) and Id-1, previously reported to be regulated by LANA [66], [67], also contained LANA peaks within promoters in both cell types.

Gene Ontology (GO) analysis was performed by using DAVID (Table S4 and S5). Interestingly, albeit the low overlap between both cell types, the two most enriched GO terms for both gene lists were chromosome organization and regulation of apoptosis. LANA binding was observed within promoters of several histone gene variants, explaining the association with chromosome organization, although coverage was stronger in TIVE-LTC. In BCBL-1 cells, putative LANA targets are related to phosphorus metabolic processes, regulation of cellular enzymatic activity, and regulation of cellular response to stress; while in TIVE-LTC cells, putative LANA targets are involved in regulation of macromolecule metabolic process, nutrient levels, and angiogenesis, the latter a hallmark of KS.

**Figure 8:**
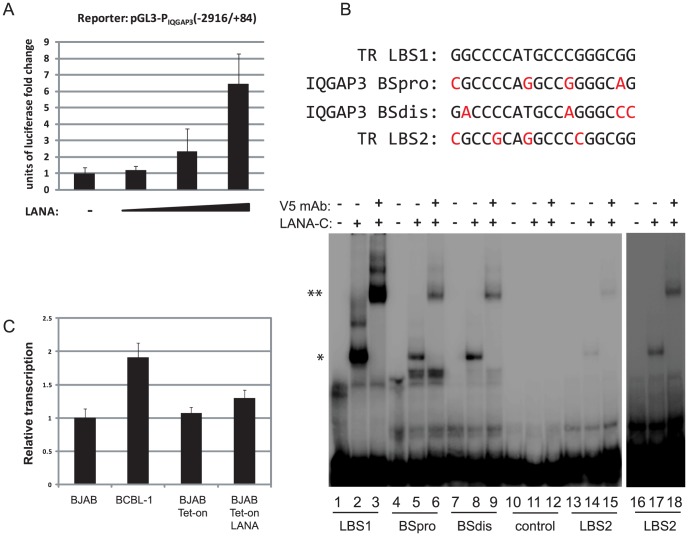
LANA binds to two sites within the IQGAP3 promoter and upregulates IQGAP3 expression. (A) Using transient transfection assays LANA upregulates IQGAP3 promoter in a dose-dependent manner. (B) The proximal and distal similar LANA binding sites form complexes with purified LANA-C protein. At the top the sequences are shown for the high-affinity LBS1 site, the low affinity LBS2 site, and the BSpro and BSdis regions of IQGAP3. Bases differing from LBS1 are shown in red. Below are the result of gel shift analysis. Lane 1, LBS1 probe; Lane 2, LBS1 probe incubated with V5-tagged LANA-C; Lane 3, LBS1 probe incubated with V5-tagged LANA-C and V5 monoclonal antibody; Lanes 4 to 6, and 7 to 9, same incubations with BSpro and BSdis from IQGAP3 promoter; Lanes 10 to 12, and 13 to 15, same incubations with either negative control fragment, or LBS2, the low affinity LANA binding site from TR. Lanes 16 to 18, longer exposure of lanes 13 to 15. Complexes with LANA-C are indicated by single arrowheads, and supershifted complexes with LANA-C and V5 mAb by double arrowheads. (C) Transcription level of IQGAP3 is induced in cells expressing LANA. LANA/Tet-on cells [53] were treated with doxycycline and total RNA extracted was analyzed by real-time RT-PCR using IQGAP3-specific primers.

Recently, Lu et al. performed LANA ChIP-seq in BCBL-1 cells and reported 256 enriched genes [58] and comparison of both data sets gained 15 genes in common including FBXO4, PARL, and IQGAP3. For functional validation, we chose IQGAP3 (IQ motif containing GTPase-activating protein 3), a regulator of cell proliferation in the Ras/ERK signaling pathway [68]. IQGAP3 was the third highest coverage LANA-binding peak in BCBL-1 cells (Table 3), and observed peaks upstream of the TSS contain two sites with homology to LANA binding sites. A proximal binding site (BSpro) is located at −90 and a distal (BSdis) is at −700 from the TSS, and both have 4 nts difference compared to the high affinity LBS1 site within the TR (Fig. 8B). A 3 Kb (−2916 to +84) promoter region of IQGAP3 was cloned upstream of a luciferase reporter and co-transfected with a LANA expression vector into HEK293 cells. As shown in Figure 8A, LANA transactivates the IQGAP3 promoter in a dose-dependent manner. Next, putative LANA binding sites were tested in EMSA assays using the C-terminal DNA binding domain of LANA (LANA-C). Mobility of both BSpro- and BSdis-containing probes was retarded in the presence of V5-tagged LANA-C (Fig. 8B lanes 5 and 8). Adding V5 mAb resulted in supershifting of the complexes of LANA-C with BSdis and with BSpro (Fig. 8B, lanes 6 and 9). Although the intensity of the LANA-C complexes with BSpro and BSdis were less than with LBS1, the complexes were stronger than seen for the low affinity LBS2 site (Fig. 8B). No complex was seen when LANA-C was incubated with a control DNA probe derived from a 38 bp portion of IQGAP3 lacking LBS-like sequences, even with prolonged gel exposure. The IQGAP3 sequence tested by Lu et al. did not contain BSpro or BSdis, and did not compete with an LBS1/2 probe for binding to LANA [58].

**Table 3:** The top 20 annotated cellular genes with LANA association within the promoter region in BCBL-1 cells and TIVE-LTC cells.

	BCBL-1 cells			TIVE-LTC cells		
	Gene name	Average Coverage	aSimilar LBS	Gene name	Average Coverage	Similar LBS
**1**	PARL	107.14546		HIST2H2BE, HIST2H2AC, HIST2H2AB	63.09397	
**2**	NIPAL2	78.05229	Yes	HIST1H1C	49.09903	
**3**	IQGAP3	65.67206	Yes	HIST1H2BJ, HIST1H2AE	44.54586	
**4**	VWA2	64.48265	Yes	MRPL53	37.97208	
**5**	KIAA1609	44.93211		HIST1H2BC, HIST1H2AC	37.76241	
**6**	PAPSS2	37.02512		NFYC	37.14510	
**7**	PINK1	34.40807	Yes	WDR74	30.00280	
**8**	PSMD9	31.78383		C8orf37	29.90638	
**9**	HECTD2	30.88176	Yes	POLR3E	27.29477	
**10**	F8A3, H2AFB3	28.97898		CCDC90B	24.47793	
**11**	SGMS1	27.40401	Yes	PLEKHG2	22.52469	
**12**	LRRTM3	24.17962		B3GNT1	22.06739	
**13**	ADSSL1	22.75948	Yes	EIF1	20.28284	
**14**	DNMT3A	22.52345		GABPA, ATP5J	18.96609	
**15**	CLTB	22.47465	Yes	ADSS	17.88085	
**16**	SPRYD3	21.61213		TUBA1B	17.38146	
**17**	C16orf86, C16orf48	20.99895	Yes	C3orf31	16.61529	
**18**	ATG14	20.62600		HIST1H1E, HIST1H2BD	15.92253	
**19**	BIRC6	20.58484		ZNFX1	15.17040	
**20**	PCDHB3	19.20834		TSEN2	14.26445	

a Similar LBS indicates motifs that align with LBS1/2 allowing up to 4 mismatches.

To test LANA regulation of IQGAP3 in cells, we determined IQGAP3 transcript levels in BCBL-1 and LANA-inducible BJAB cells. IQGAP3 mRNA levels are about 2-fold higher in BCBL-1 cells and induction of LANA in BJAB-Tet on-LANA cells moderately induced IQGAP3 transcription (Fig. 8C). Together these data demonstrate that LANA can directly bind and positively regulate the IQGAP3 promoter. Although demonstrated on a single promoter, these data further validate potential LANA targets identified by ChIP-seq, and suggest that LANA contributes to the regulation of a subset of these genes.

### Identification of a novel LANA binding motif

**Figure 9:**
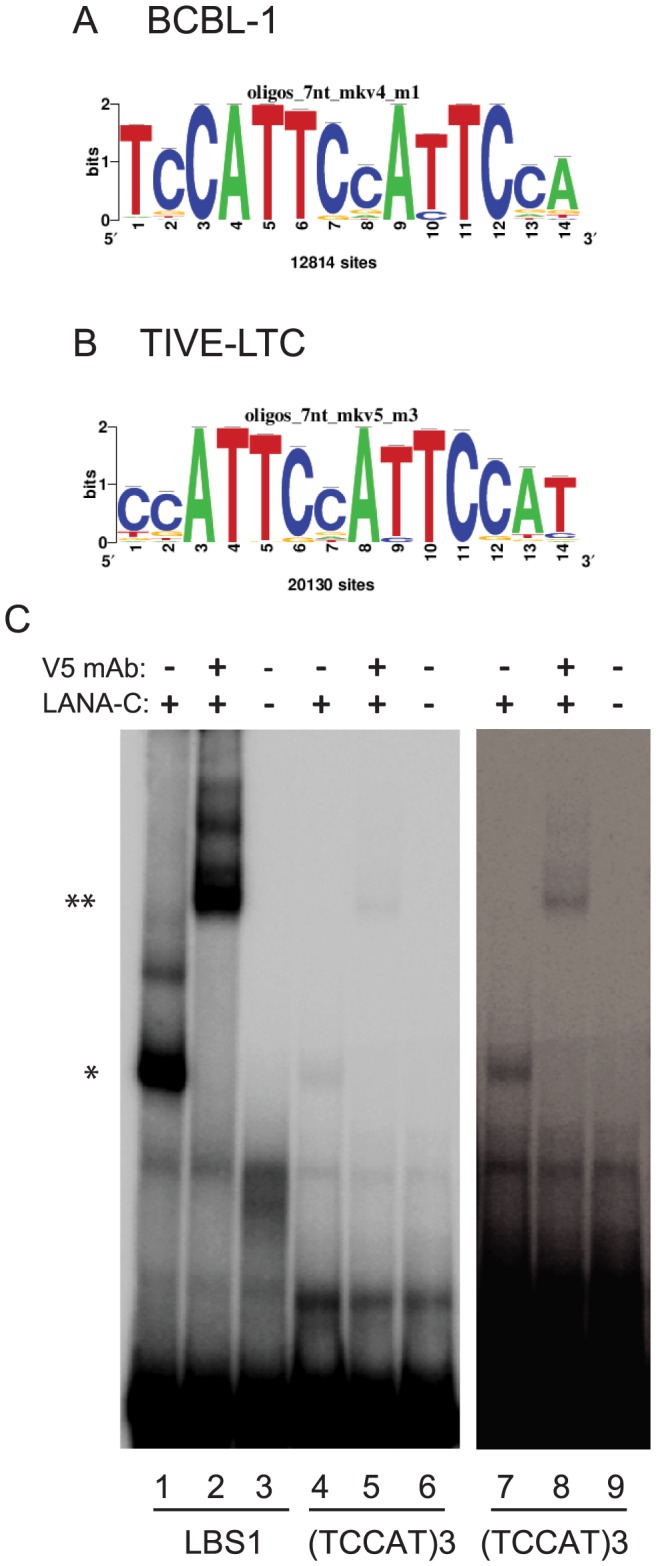
Identification and biochemical characterization of novel LANA binding motif. Peak Motif analysis of LANA enriched sequences retrieved to highly homologous consensus sequences from BCBL-1 (A) and LTC-TIVE (B) cells. (C) LANA-C binds to a novel sequence motif. Lane 1, LBS1 probe incubated with V5-tagged LANA-C; Lane 2, LBS1 probe incubated with V5-tagged LANA-C and V5 monoclonal antibody; Lane 3, LBS1 probe. Lanes 4 to 6, and 7 to 9, same incubations with (TCCAT)3 probe. Lanes 7 to 9 longer exposure to visualize bands. Comparison to Fig. 8 shows similar binding to LBS2.

LANA peaks were screened for LBS1/2 consensus sites allowing up to four mismatches. In BCBL-1, 58 out of 2180 (2.7%) and in TIVE-LTC cells 205 out of 2951 (6.9%) peaks contained sequence similarity to LBS1/2 (Fig. S5). Hence, some enhancers/promoters may be bound directly by LANA while the majority of LANA peaks result either from protein/protein interaction or from binding unidentified sequence-specificities. To identify potentially novel LANA binding motifs, all DNA sequences enriched by LANA in BCBL-1 and TIVE-LTC cells were analyzed for consensus sequences using “peak motifs” from Regulatory Sequence Analysis Tools (RSAT) [69], [70]. In BCBL-1 cells, 12,814 sites contained a unique 14-nts long motif (Fig. 9A). Coverage of three additional motifs was significantly lower (<3500) but contained a similar core sequence. Significantly, in TIVE-LTC cells 20,130 sites contained a motif very similar (13/14) to the one observed in BCBL-1 (Fig. 9B). These results suggest that LANA either directly binds to the motif or associates with other proteins bound to the motif. We searched the known transcription factor binding sites with this motif in the JASPAR database, but failed to identify any known transcription factor with this motif. A single consensus motif without homology to known transcription factor binding sites derived from ChIP-data from two different cell types may point to a novel LANA binding specificity or alternatively, a non-characterized LANA/DNA binding protein interaction. The 15 base sequence (TCCAT)_3_ formed from overlapping the motifs in Fig. 9AB was tested for binding by LANA-C using EMSA (Fig. 9C). The DNA probe containing the (TCCAT)_3_ motif formed a complex with LANA-C that was visible on longer exposures comparable to those used to detect binding of LANA-C to LBS2. The complex was supershifted in the presence of V5 mAb, confirming specificity. This demonstrates that the 15 nt (TCCAT)_3_ sequence (Fig. 9AB) present in high copy numbers in the human genome is a novel LANA binding motif whose affinity is comparable to LBS2.

### LANA co-occupancy with host transcription factors at cellular promoters

**Figure 10:**
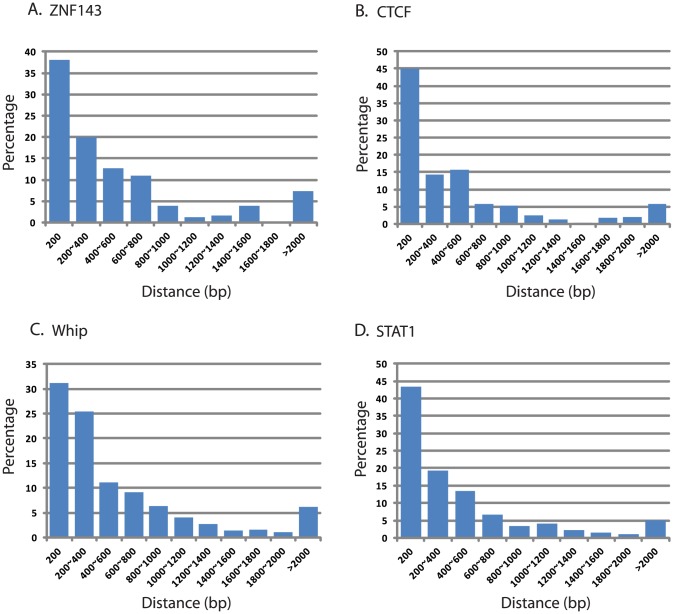
Distance of LANA peaks and four transcription factor binding site at LANA-bound promoter regions using ENCODE data. Distance was plotted based on analysis of ENCODE transcription factor ChIP-seq data set for GM12878, a B cell lymphoma: (A) ZNF143, (B) CTCF, (C) Whip, and (D) STAT1. Between 32% (Whip) and 45% (CTCF) of LANA binding peaks are located within 200 bp of their respective transcription factor binding sites.

Next, we asked whether LANA-bound promoters were enriched for specific host transcription factors. Genome-wide occupancy data for many transcription factors are available through the Encyclopedia of DNA Element (ENCODE) Consortium. As the closest available cell line to BCBL-1, we mined ChIP-seq data from GM12878 cells, a B cell lymphoma cell line, for which 43 genome-wide transcription factor binding profiles are available [71]. Since ENCODE contains only six datasets for endothelial cells, this analysis was not performed in TIVE-LTC. ENCODE GM12878 ChIP-Seq data were mapped to the hg19 promoter regions, and compared to LANA occupancy observed in BCBL-1 cells. Transcription factor and LANA peaks within 2 kbp from the TSS were analyzed; furthermore, we calculated and plotted the distance distribution of these peaks. Table 4 lists the number of individual transcription factors peaks co-present at putative LANA regulated promoters, and tabulates the percentage of genes where co-occupancy is predicted. Interestingly, between 83% and 88% of the 167 LANA-binding promoters identified in BCBL-1 contain ZNF143, CTCF, Whip, STAT1, or ebf1 binding peaks (Table 4). Both LANA and CTCF, which contribute to latent and lytic gene expression, co-occupy the LANA and RTA genes within the viral genomes [36], [60], [61]. Distance analysis showed that 45% of all LANA peaks are within 200 bps and 60% are within 400 bp of CTCF sites, which expands a role for co-regulation of LANA and CTCF to host genes (Fig. 10B). Similarly, 65% of LANA peaks were within 400 bps of STAT1 binding sites (Fig. 10D). Hence, LANA binding may modulate promoters regulated by STAT1, a master transcriptional regulator of immunity, cell cycle, and proliferation [72]. While many of the LANA peaks are within 200 bps of STAT1 binding sites, we did not observe LANA binding overlapping STAT1 sites, as was previously reported [58]. Co-occupancy of LANA with transcriptional regulators ZNF143, a strong regulator of cell cycle control and proliferation (Fig. 10A), whip, a transcription factor involved in DNA damage (Fig. 10C), and ebf1, a B cell-specific transcription factor (Table 4), suggest that LANA binding may affect multiple complex regulatory pathways in latently infected cells. The fact that other transcription factors like the ubiquitously expressed zinc finger protein Ying Yang 1 (YY1) were not enriched suggests specificity, which is further supported by the cell type specificity of the observed LANA-bound promoters.

**Table 4:** Co-occupancy of LANA and transcription factors at cellular promoters.

Transcription Factor	LANA peak #	aTF peak #	Transcripts Affected		Gene Symbol Affected	
			number	% of total	number	% of total
**znf143**	311	462	1080	83.40	148	88.62
**CTCF**	296	392	1044	80.62	145	86.83
**whip**	293	493	1047	80.85	140	83.83
**STAT1**	274	404	974	75.21	137	82.04
**ebf1**	270	444	986	76.14	139	83.23
**maz**	263	341	954	73.67	133	79.64
**smc3**	257	334	882	68.11	120	71.86
**bhlhe40**	249	321	922	71.20	131	78.44
**mxi1**	246	325	922	71.20	134	80.24
**max**	241	335	939	72.51	140	83.83
**corest**	238	310	918	70.89	130	77.84
**tblr1**	235	300	898	69.34	133	79.64
**chd2**	231	297	904	69.81	133	79.64
**chd1**	215	292	851	65.71	124	74.25
**p300**	202	259	775	59.85	105	62.87
**tbp**	202	255	807	62.32	122	73.05
**elk1**	201	266	804	62.08	112	67.07
**rfx5**	190	232	772	59.61	113	67.66
**sin3a**	188	227	791	61.08	122	73.05
**nrf1**	170	205	767	59.23	112	67.07
**usf2**	162	185	650	50.19	95	56.89
**brca1**	160	184	621	47.95	91	54.49
**cdp**	151	188	591	45.64	72	43.11
**stat3**	133	152	484	37.37	66	39.52
**rad21**	122	130	370	28.57	45	26.95
**cmyc**	111	202	484	37.37	19	11.38
**nfyb**	111	129	446	34.44	72	43.11
**ikzf1**	107	114	267	20.62	38	22.75
**e2f4**	104	102	465	35.91	62	37.13
**irf3**	101	113	373	28.80	51	30.54
**nfe2**	99	118	353	27.26	57	34.13
**gcn5**	68	73	215	16.60	33	19.76
**spt20**	50	54	157	12.12	17	10.18
**nfya**	46	44	156	12.05	27	16.17
**esrra**	30	29	89	6.87	13	7.78
**srebp1**	26	24	117	9.03	14	8.38
**znf274**	20	19	22	1.70	1	0.60
**jund**	19	18	45	3.47	10	5.99
**srebp2**	15	15	49	3.78	5	2.99
**cfos**	13	12	53	4.09	11	6.59
**tr4**	10	9	63	4.86	10	5.99
**YY1**	7	7	43	3.32	7	4.19
**MAFK**	4	4	9	0.69	1	0.60

a ENCODE GM12878 ChIP-Seq data were mapped to the hg19 promoter regions, and compared to LANA occupancy observed by ChIPseq in BCBL-1 cells.

### LANA predominantly binds to H3K4me3 decorated promoters and associates with hSET1 complexes

**Figure 11:**
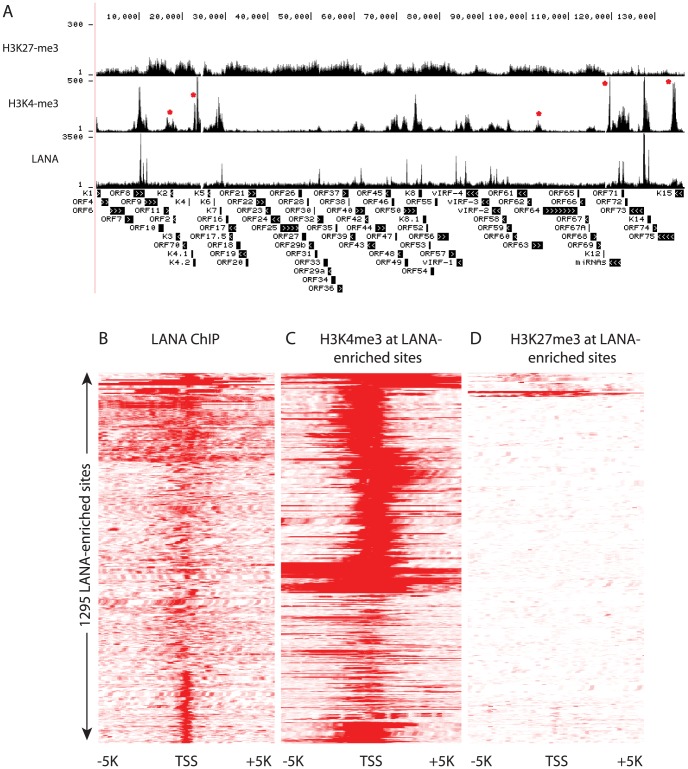
LANA and H3K4me3 but not H3K27 overlap at many regions of the KSHV and human genome in BCBL-1 cells. (**A**) ChIP-seq was performed in BCBL-1, with LANA-, H3K4me3-, H3K27me3-specific antibodies. Sequencing tags were mapped to viral genome and visualized in UCSC Genome Browser. Cluster analysis (seqMINER) using sequence regions (plus/minus 5 kbp around TSSs) that were enriched by LANA ChIP-seq upstream of 1295 annotated transcripts in BCBL-1 cells (Table 2 and Table S1). (B) Heterogeneous LANA binding patterns relative to TSSs; (C) H3K4me3 distribution at LANA-enriched sites and (D) H3K27me3 distribution at LANA-enriched sites.

Comparison of H3K4me3 and H3K27me3 marks to LANA peaks on viral episomes revealed regions that were enriched for LANA peaks lacked H3K27me3, but in most cases contained H3K4me3 peaks. On the viral genome H3K4me3 is enriched at all observed LANA peaks (Fig. 11A); however, there are five regions where H3K4me3 peaks are present in the absence of LANA: 1) a wide peak from nt 16 K to nt 18 K spanning ORF11, K2 and ORF2; 2) two sharp peaks up-stream of K4.2; 3) a minor peak at nt 103 K within the coding region of ORF63; 4) a major peak downstream of the miRNA cluster (nt 119,500); and 5) within the K15 gene. Additionally, these regions are void of Pol II (Figs. 1 and 4), which is characteristic of enhancers that can be enriched for H3K4 methylation but lack Pol II [1], [2]. These patterns suggest that LANA predominantly binds to active promoters. We extended this analysis to the human genome hg19 and determined that in both BCBL-1 and TIVE-LTC cells, a strong relationship exists between LANA binding at promoters and H3K4me3 (R^2^ = 0.9) (Fig. 11 BC). This suggests that LANA predominantly plays a role as a positive regulator of gene expression. To further address this, we analyzed expression patterns of the 167 genes that showed promoter-associated LANA peaks, by analyzing previously published profiling data [73], [74] and found that 86% of these genes are indeed expressed in BCBL-1 cells. In summary, these data demonstrate that LANA preferentially associates with promoters that carry H3K4me3 marks and are transcriptionally active.

**Figure 12:**
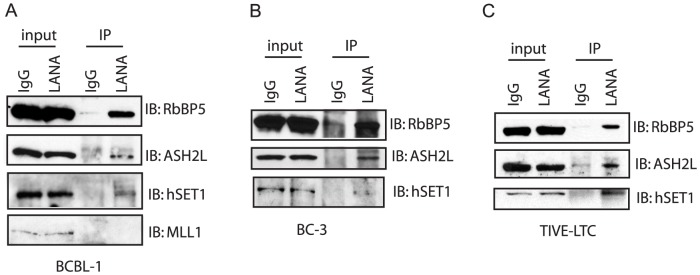
LANA forms complex with H3K4me3 methyltransferase hSET1 *in vivo*. Nuclear extract was harvested from BCBL-1 cells (A) or BC-3 cells (B) and LTC-TIVE (C). The nuclear extract was immunoprecipitated with control IgG or monoclonal rat antibody against LANA. The immunoprecipitated complex was separated in 8% SDS-PAGE gel and immunoblotted with RbBP5, ASH2L, and hSET1, or MLL-1 antibody.

The strong correlation of LANA and H3K4me3 peaks (Fig. 11) raised the possibility that LANA may play a role in the methylation of histone H3K4, which may contribute to the establishment and maintenance of latency by preventing H3K27me3-dependent silencing of latency-associated promoters. To address this question, we performed immunoprecipitation assays in BCBL-1 cells to determine whether LANA associates with histone H3K4 methyltransferases. In mammalian cells a number of different H3K4 lysine methyltransferases (KMTs) exist that function mostly non-redundantly. The MLL/SET1 family, including MLL1-5 and hSET1, are the major methyltransferases. Members in this family are multi-subunit complexes that share a core complex composed of three proteins: RbBP5, ASH2L, and WDR5 [3], [4], [5]. Accordingly, BCBL-1 cell lysates were immunoprecipitated with LANA-specific monoclonal antibody, and precipitated protein complexes were assayed for the presence of the endogenous MLL1-5/SET1 family core proteins by Western blotting. As shown in Figure 12A, LANA co-precipitated with RbBP5 and ASH2L, the core components of MLL/hSET1 family KMTs. We detected LANA interaction with hSET1, but not MLL1, in BCBL-1 cells, which express low levels of MLL1. The association with both the hSET1 complex core proteins RbBP5 and ASH2L and hSET1 itself was further confirmed in a second PEL cell line (BC-3) and in latently infected endothelial cells (TIVE-LTC) (Fig. 12BC). To ask whether LANA directly interacts with the hSET1 core proteins we performed GST pull-down assays with purified GST-ASH2L, GST-WDR5, or GST-RbBP5 and full-length *in vitro* translated LANA, but did not detect direct interaction with these proteins (data not shown). In summary, these data show that LANA interacts with endogenous hSET1 complexes either directly or through protein-protein interaction. Recruitment of hSET1 complexes to specific chromatin loci has been reported to be mediated by a number of transcription factors or co-activators, including E2F, NF-E2, and USF1 [3], [4], [5]. The HSV-1 VP16 protein recruits H3K4me3 KMTs to immediate early promoters after *de novo* infection by interacting with HCF-1, which subsequently binds to and recruits hSET1 [12], [13].

## Discussion

Previous reports by several independent groups demonstrated the importance of H3K27me3 deposition for both the establishment and maintenance of KSHV latency [34], [35], [55]. Decreases of H3K27me3 by either overexpression of JMJD3, the H3K27 demethylase, or by blocking with small molecule inhibitors the H3K27 methyltransferase EZH2, disrupt latency and induce KSHV reactivation. Very recently, Toth et al. demonstrated that the deposition of H3K27me3 follows after an initial phase of lytic gene expression associated with H3K4me3 deposition [75]. Hence, KSHV latency and reactivation seem to be largely controlled by a balance between H3K27me3 and H3K4me3 deposition to specific genomic regions. Recently, LANA was demonstrated to interact with and recruit KDM3A, which demethylates H3K9me1/2, a mark associated with heterochromatin. While inhibiting KDM3A affected the extent of lytic replication after induction, overexpression of KDM3A, unlike JMJD3, did not induce reactivation [22]. However, very little is known about the potential mechanisms by which viral proteins prevent PRC2 complexes from silencing KSHV after *de novo* infection or during long-term latency.

### Epigenetic profiles of the viral episomes are similar in lymphoid and endothelial cells

As with two previous studies, we found that in BCBL-1 latency-associated genes are enriched with H3K4me3 and PolII but depleted of H3K27me3 (reviewed in [55]). A number of lytic genes including ORF9, K4.2, K7, K8, and ORF58 also contain H3K4me3 marks. Strong Pol II peaks and H3K4me3 were detected next to the OriLytL and at the TSS of lytic genes K7, and within the K5 and K6 ORFs. These data are in congruence with Toth et al., which demonstrated that Pol II transcription of these genes is stalled by association with cellular negative elongation factor NELF [51]. The H3K4me3 deposition pattern was largely identical in TIVE-LTC, which provided the first genome-wide epigenetic analysis of latently-infected endothelial cells. Interestingly, the enrichment of Pol II at oriLyt region was undetectable in TIVE-LTC cells which cannot be efficiently reactivated in culture [53] (Fig. 4), suggesting that bivalent marks on promoters other than RTA contribute to efficient reactivation.

Previously, Chandriani and Ganem performed transcript profiling by limiting dilution PCR during latency in BCBL-1, SLK.219, and HFF.219 cells and identified vIL-6 expression during latency [50]. We identified both PolII and H3K4me3 on the vIL6 promoter (K2), in both BCBL-1 and TIVE-LTC cells. The latter cell line is strictly latent and therefore provides evidence for vIL6 transcription during latency in endothelial cells. These data are further supported by recent studies on chromatin structure, which identified a nucleosome-free region at the vIL6 promoter in latently infected BCBL-1 and LTC-TIVE cells [49], [76].

### LANA binds to viral genes of all kinetic classes and potentially contributes to regulation

LANA ChIP-seq from both cell lines identified 17 highly reproducible LANA peaks on the viral genome (Table 1 and Figure 5). LANA binding patterns are very similar between cells of lymphoid and endothelial origin (Figure 5). The highest RPKM coverage was seen on the TR where LANA bound at two LANA binding sites (LBS1/2) [20]. In addition to the previously described LANA and RTA signals, we detected LANA binding upstream of IE, E, and late genes (Table 1). We showed that at least in the context of reporter assays the promoters of ORF16, 39, 48, and vIRF1 all responded to LANA *in trans*. These data are congruent with previous data showing that LANA can augment transcription from a wide range of promoters [24], [57], [59]. A potential role for LANA during reactivation would explain the presence of a second RTA-responsive promoter upstream of the LANA ORF [45]. However, such a role would have to be early since Kim et al. demonstrated that LANA association with viral episomes decreased at about 4 hours post reactivation [22]. Experiments interrogating whether LANA modulates lytic gene expression in the context of reactivation are currently ongoing. Lu et al. reported LANA binding on viral and human genomes in BCBL-1 cells [58]. While both studies utilized an identical antibody and cell line (BCBL-1 cells), Lu reported only two low coverage peaks at the K7 and vIRF2 regions outside of the TR, which were detected in our study; differences with respect to coverage of the viral genome may largely result from usage of different sequencing methods.

### LANA associates with many promoters of cellular genes that are enriched for H3K4me3 and are actively transcribed

LANA binds close to TSS of H3K4me3 decorated promoters, and often co-occupies with transcription factors and the boundary element CTCF (Fig. 7, 10 and Table 4). LANA binding to host genes is cell type-specific with more promoters bound in endothelial cells. A small number of LANA enriched sequence tags showed sequence homology to the consensus LANA binding sites (58 of 2180 in BCBL-1 and 205 of 2951 in TIVE-LTC) (Table 1). RSAT motif analysis [70], [77] revealed a common consensus sequence (CCATTCCATTCCA) that is highly prevalent in the human genome and was highly enriched in both cell types (Fig. 9 AB). EMSA and supershift analysis demonstrated direct LANA binding to this novel motif (Fig. 9C). The affinity of LANA-C for the (TCCAT)_3_ motif was lower than LBS1 but comparable to LBS2, the low affinity site within the TR [20]. The existence of thousands of copies of this motif, many of which are within repeats (data not shown), suggests that this binding may be biologically significant. In fact, episomal tethering to the chromosome via binding between the C-terminus of LANA and this novel motif is consistent with reports indicating that the C-terminal DNA binding domain of LANA contributes to chromosome binding [78], [79], [80]. In summary, the binding of LANA-C to LBS1/2-like sequences and the novel (TCCAT)_3_ consensus sequence demonstrates that LANA can directly bind to host cellular DNA via two distinct sequence motifs. This interpretation is also supported by cluster analysis using seqMiner algorithms, which revealed a number of distinct binding patterns across all LANA-enriched promoters (Fig. 11B).

Mercier et al. [81] recently reported a LANA ChIP-seq analysis performed in PEL cells and in lymphatic endothelial cells (LEC), which were previously shown to display a unique gene expression profile that is markedly different from latency [82]. Several findings agree between both studies, specifically the fact that many more promoters showed LANA binding in endothelial cells versus lymphoid cells and that these were largely cell type specific. Moreover, the findings by both groups that LANA binds close to TSS that are decorated with H3K4me3 and actively transcribed are in agreement. In addition, Mercier et al. performed RNA-Seq experiments in uninfected and KSHV-infected LEC cells and showed that only a small number of host genes bound by LANA were differentially expressed [81]. This is consistent with our observation that numerous viral genes and 14% of cellular promoters that are bound by LANA are not expressed during latency in BCBL-1 cells (Fig. 1, 4). Hence, LANA binding alone does not induce transcription in the context of chromatin. LANA may act at the epigenetic level by influencing histone modifications. Alternatively, LANA may merely have a higher propensity to bind to H3K4me3 decorated promoters since they are often transcribed and contain open chromatin. However, as demonstrated here for the IQGAP3 gene (Fig. 8), and by numerous previous studies, host cellular genes can be directly regulated by LANA [66], [67], [74]. With respect to analyzing how LANA binds to DNA, our studies differ in the proportion of LANA peaks that contain LBS1/2-like sequences. Mercier identified a sequence nearly identical to LBS1 in 157/267 (58.8%) of the LANA ChIP peaks, but we found 58/2180 (2.7%) of peaks from BCBL-1 cells contained sequences resembling LBS1, and 205/2951 (6.9%) from TIVE-LTC cells. These differences may result from using different bioinformatics tools in the two studies. Additionally, we identified and biochemically characterized a novel LANA-binding sequence motif (TCCAT)_3_, which occurs with high frequency in the human genome.

### LANA associates with hSET1 complexes in cells of lymphoid and endothelial origin

Motivated by LANA's preferential association with H3K4me3 mark-containing promoters, we asked whether LANA interacts with KMTs and demonstrated that LANA efficiently immunoprecipitates with hSET1, the main H3K4 methylase in mammalian cells [3], [4] (Fig. 12). We did not detect direct binding to the hSET1 core components RbBP5, WDR5, and ASH2L [5], [6], [7] and do not know whether LANA interacts with any of the remaining hSET1 components or through a bridging factor. A detailed biochemical and genetic study to determine how LANA interacts with and potentially modulates H3K4me3 deposition is currently ongoing. In support of this observation, hSET1 is not the only epigenetic modifier complex shown to interact with LANA. Kim et al. recently demonstrated that LANA association with the histone demethylase KDM3A regulates viral gene expression during both latent and lytic replication [22]. While the LANA-hSET1 interaction is novel for γ-herpesviruses, the HSV-1 VP16 protein is known to recruit hSET1 and MLL complexes to immediate early promoters through an interaction with HCF-1 [12], [13].

We propose a working model for the establishment of the viral epigenome which integrates recent findings affecting i) the epigenetic variation of KSHV episomes [49], [76], and ii) novel mechanistic insights into how PRC2 deposits H3K27me3 marks [83].

After *de novo* infection an early burst of promiscuous transcription which includes both LANA and the RTA gene occurs which leads to co-transcriptional H3K4me3 deposition at many promoters [1], [75], [84]. We envision that LANA is recruited to many promoters that are initially active through an hSET1-dependent mechanism. As a result PRC2-dependent silencing is stopped at regions where LANA is bound and H3K4me3 has been deposited. Recent chromatin structure mapping analysis on the LANA, RTA, and vIL6 promoters demonstrated that a subpopulation of episomes in PEL cells carry nucleosome free regions (NFRs) [49], [76], which recently have been shown to prevent H3K27me3 marks from spreading [83]. Moreover, these NFRs are flanked by CTCF boundary elements as in the LANA promoter [36], [37], [60], [61], [85]; additionally LANA binding sites were highly correlated with CTCF binding on both the viral and host chromatin (Table 4).

We envisage competition between PRC2 silencing [34], [35] and LANA recruitment of KDM3A [22] and possibly hSET1 to form euchromatin on a number of latent promoters as well as promoters essential for reactivation. As a result a small number of episomes will carry epigenetic marks that are “permissive for latency”. Conversely, another subpopulation of episomes will be completely silenced by host-dependent heterochromatin formation and as a result will neither contribute to latent nor lytic gene expression, as recently suggested in BCBL-1 and TIVE-LTC cells by single copy chromatin mapping [49]. Further understanding the precise molecular mechanisms by which LANA contributes to maintenance of euchromatin may yield approaches to tip the balance towards complete epigenetic silencing as a novel intervention strategy.

## Materials and Methods

### Cell lines

293 cells, the human embryonic kidney cells, and KSHV long term-infected telomerase-immortalized human umbilical vein endothelial cells (TIVE-LTC) [53] were cultured in Dulbecco's modified eagle medium (DMEM) supplemented with 10% fetal calf serum (FCS) and antibiotics at 37°C under 5% CO_2_ atmosphere. BCBL-1 and BJAB cells were cultured in RPMI 1640 medium supplemented with 10% FCS and antibiotics at 37°C under 5% CO_2_ atmosphere.

### Plasmids

Primer pairs were designed to amplify the promoter region (+/−2 kb relative to the transcription start site) for IQGAP3 (5′-GATCGGTACCACAACCCAGTCTCTAAACCAG-3′ and 5′-GACTACGCGTGTTCCTTAGGCTGCCCC), ORF16 (5′- CCGCTCGAGCGGTTGTCAACCAACCAGTCAATCACC and 5′- CCCAAGCTTGGGGCAAAACGTCCTCGTCCATT), ORF39 (5′- GGGGTACCCCGAATGATGTTTGTCTTCGCC and 5′- CCGCTCGAGCGGCGCATGTTTCTCGGTCTTTT), ORF48 (5′- GGGGTACCCCTCTACCATGGAAGCCGGCAA and 5′- CCGCTCGAGCGGGGATACACACCTCCATGTTC), and vIRF1 (5′- GGGGTACCCCATGGAAGCCGGCAACAGTCCT and 5′- CCGCTCGAGCGGACACCTCCATGTTCAGTCAC). Corresponding promoter regions were PCR amplified, and cloned into plasmid pGL3-basic at appropriate sites.

### Antibodies

Antibodies used are control rabbit IgG (Santa Cruz Biotechnology, sc-2027), rat IgG (Santa Cruz Biotechnology, sc-2026), rabbit against H3K4me3 (Abcam, ab8580), H3K27me3 (Upstate, 07-449), rabbit anti Pol II (Santa Cruz Biotechnology, sc-899), rat LANA monoclonal antibody (ABI Inc., LN53), rabbit anti hSET1 (Bethyl, A300-289A), rabbit anti RbBP5 (Bethyl, A300-109A), rabbit anti ASH2L (Bethyl, A300-489), and rabbit anti MLL1 (Upstate, ABE240).

### Chromatin immunoprecipitation (ChIP)

ChIP experiments were performed as described before with minor modifications [32]. BCBL-1 or TIVE-LTC cells were crosslinked with 1% formaldehyde at room temperature for 10 min. Crosslinking was terminated by adding glycine to a final concentration of 0.125M. Cells were washed twice in ice-cold PBS with protease inhibitors and harvested by centrifugation. Every 2×10^7^ cells were lysed in 1 ml ice-cold Farnham lysis buffer (5 mM PIPES [pH 8.0], 85 mM KCl, 0.5% NP-40) with protease inhibitors. The nuclei were spun down and resuspended in 1 ml RIPA buffer (1% NP-40, 0.5% sodium deoxycholate, and 0.1% SDS in 1×PBS) with protease inhibitors. Chromatin was sheared to about 250 bp fragments with 5 sets of 30-second pulses using a Sonic Dismembrator (Fisher Scientific) set to 50% of maximum power. Chromatin from 6×10^7^ cells was incubated with 10 µg primary antibody (normal rabbit IgG, rabbit anti H3K4me3, rabbit anti H3K27me3, normal rat IgG, or rat anti LANA) and 100 µl magnetic beads (sheep anti rabbit-conjugated, or protein A-conjugated) at 4°C overnight on a rotator. Beads were washed 5 times with LiCl wash buffer and once with TE. The immune complexes were eluted with 200 µl elution buffer twice at 65°C for 1 hour. The combined eluates were de-crosslinked at 65°C overnight. DNA was extracted once with phenol/chloroform and precipitated with ethanol. 20 µg glycogen was added as DNA carrier. DNA pellets were washed once with 70% ethanol and resuspended in 40 µl H_2_O.

### Library construction for Illumina sequencing

100 ng ChIP-enriched DNA or control IgG ChIP DNA was blunt-ended with T4 DNA polymerase and Klenow DNA polymerase (NEB) and phosphorylated with T4 PNK (NEB). Addition of an “A” base to the 3′ ends of the blunt phosphorylated DNA fragments was performed in the presence of Klenow exo- (NEB). Subsequently, adapters (5′-pGATCGGAAGAGCGGTTCAGCAGGAATGCCGAG and 5′- ACACTCTTTCCCTACACGACGCTCTTCCGATCT) were ligated to both ends of the DNA fragments. DNA within the range of 150 bp to 300 bp was gel-purified and PCR-amplified for 18 cycles with primer 5′ -AATGATACGGCGACCACCGAGATCTACACTCTTTCCCTACACGACGCTCTTCCGATCT and 5′- CAAGCAGAAGACGGCATACGAGATCGGTCTCGGCATTCCTGCTGAACCGCTCTTCCGATCT. The resulting libraries were gel-purified, quantified with QuantIT dsDNA Assay Kit (Invitrogen) and sequenced with Genome Analyzer IIx (Illumina) in Department of Molecular Genetics and Microbiology (MGM) at University of Florida (UF).

### Enrichment of TIVE-LTC ChIP-seq libraries with SureSelect System

The biotin-labeled RNA baits specific for KSHV genome was customized with eArray XD (Agilent) with help from Agilent. The RNA baits are 120 nt long with 4× tiling frequency. TIVE-LTC ChIP-seq libraries were constructed as above. After adapter ligation, DNA fragments between 150 bp and 300 bp were gel-selected and amplified with 10 cycles in first PCR. Samples were purified with Agencourt AMPure XP beads (Beckman Coulter). The KSHV-specific DNA sequences were enriched with SureSelect Enrichment System (Agilent) according to the manufacturer's instruction. The purified 1^st^ PCR products were denatured and hybridized with KSHV RNA baits at 65°C for 48 hours in PCR machine with heated lid. The RNA-DNA hybrids were recovered with Dynal MyOne Streptavidin T1 magnetic beads (Invitrogen). The captured DNA was eluted and purified. The DNA was re-amplified for 22 PCR cycles with primers (5′-AATGATACGGCGACCACCGAGATCTACACTCTTTCCCTACACGACGCTCTTCCGATCT and 5′-CAAGCAGAAGACGGCATACGAGCTCTTCCGATCT), and purified using Agencourt AMPure XP beads. The libraries were quantified with QuantIT dsDNA Assay Kit (Invitrogen) and sequenced as above. HTS sequencing generated 20.8 million, 17.1 million, and 20.5 million tags for H3K4me3, H3K27me3, and Pol II ChIP-seq, respectively. After enrichment 42.76%, 65.4%, and 87.58% of H3K4me3, H3K27me3, and Pol II ChIP-seq tags were mapped to the viral genome compared to 0.014%, 0.028%, and 0.007% without enrichment; hence enrichment efficiency was about 2,000- to 4,000-fold.

### Data analysis

#### Reads alignment

After completion of Illumina sequencing, the raw output of BCL files were converted to the FASTQ format using the Illumina BCL Converter. Bowtie [38], [86], a short reads aligner application, was used to first align the sequencing tags against the KSHV genome (accession number NC_009333). The unaligned tags were subsequently aligned against human genome sequence hg19. For both alignments, Bowtie was run using default settings and additional options “–tryhard –best –strata” to obtain the alignments of the best quality. The resulting alignment SAM files were converted to various formats such as BAM and MPILEUP for downstream analysis. WIGGLE and TDF files were generated for visualization in the Integrative Genomics Viewer (IGV, Broad Institute). The ChIP data have been deposited in NCBI's Gene Expression Omnibus [87] and are accessible through GEO Series accession number GSE52421 (http://www.ncbi.nlm.nih.gov/geo/query/acc.cgi?acc=GSE52421).

#### Peak finding, coverage and agreement

Peak finding was performed with CisGenome v1 [43] following the developers' manual. We used the default settings of the One-Sample Analysis. Reads from the forward and reverse strands were shifted towards fragment center by half of the estimated fragment length to refine the boundary of the binding region. Peaks on hg19 were retained if the sum of reads within a 100 bp window was greater than the cutoff value 10. This was a stringent threshold and only peaks of high confidence were included for next steps. The short KSHV genome resulted a much higher sequencing depth than that of hg19, especially in the terminal repeat (TR) region which had ∼40× more coverage compared to the rest of its genome. We determined the cutoff values for TR and non-TR regions separately based on the quantile of the sequencing depth to reflect the difference in the read count levels. These cutoff values, which were 2×90% percentile for the non-TR and 5× median for the TR respectively, were optimized to enable identification of every peak that had visible increase of reads when viewed in a genome browser. Next, peaks were compared across replicates. A peak was considered reproducible when it was identified in both replicates of the BCBL1 cells, or in at least three out of the four replicates of TIVE-LTC cells. Reproducible peaks were combined by taking the maximal overlapping regions from individual samples, and irreproducible peaks were removed. The coverage over the combined reproducible peaks quantified in reads per kilobase of peak per million mapped reads [88]. The consistency among replicate samples was demonstrated by the Bland-Altman plot [41], [42], [89], [90], where the difference between the RPKM values of two independent samples was plotted against the average. An overview of processing of all LANA ChIP-seq data from BCBL-1 and TIVE-LTC cells is given as flow diagrams in Fig. S5.

In order to examine the reads that might align to the junction of two TRs and that might be unaligned to the reference genome with only one TR at the end, we attached the sequence of a half or an entire TR to the viral genome and ran the alignment pipeline against these new reference (We did not add more TRs because repetitive sequence in the reference genome would not be aligned uniquely).

#### Correlation of H3K4me3 marks and LANA regulation

Visualization in IGV indicated that the chromatin mark H3K4me3 usually existed near LANA binding signals. We applied CisGenome on H3K4me3 ChIP-seq data but did not obtain reasonable peaks. The finding was consistent with the consensus that most peak calling programs were not designed for histone data and therefore performed poorly in identifying these diffuse signals [91]. To systematically estimate the correlation between the H3K4me3 and LANA data, we asked if there was an increase of reads in the H3K4me3 samples within the genomic regions where LANA binding peaks were identified. The coverage of H3K4me3 in the combined reproducible LANA peaks was calculated in the unit of RPKM, and compared to that of the LANA samples.

#### Promoter analysis

To study the potential regulatory function of LANA in gene expression, we mapped the reads aligned to the human genome to the promoter regions. We downloaded transcript information of Ensembl build 62 from UCSC Table Browser and defined promoters as the +/−2 Kb region of the transcription start site (TSS) of annotated transcripts. The genome positions of peaks were compared to the promoter positions using BEDTools intersectBed and any overlap was reported. The distances between the center of the peaks and their adjacent TSS's were plotted for each lane. Putative targets genes of LANA regulation were compared between BCBL1 and TIVE-LTC, as well as with previous publications.

Similarly, the reads aligned to the KSHV genome were also mapped to KSHV genes, the genome positions of which were downloaded from the NCBI Nucleotide database (NC_009333.1) and parsed using a custom Perl script.

#### LANA binding sites

The peaks were scanned for LANA consensus LANA binding sites (LBSs): GCCCCATGCCCGGGCGG (high affinity, LBS1), GCCGCAGGCCCCGGCGG (low affinity, LBS2), ATTGTCCCGGGCGCCGCG, and CCGGGTCTCCAGGGCGCGCCGCGTG. For this purpose, we used an EMBOSS tool FUZZNUC [92], which allows ambiguities in the alignment, to search for the three LBS patterns in the sequence FASTA files. FUZZNUC was run with settings to search matches from both strands and report those with four or fewer mismatches (>76% similarity). Next, we compared the genomic positions of LBS and LANA peaks, and identified LANA peaks that contained LBS within 150 bp using the BEDTools function windowBed [93].

#### Comparing to ENCODE ChIP-Seq data

To study the tissue-specific co-occupancy of LANA and other TFs, we downloaded the peak files of all ENCODE TFBS ChIP-seq experiments in GM12878 (a lymphoma cell line; comparable to BCBL1). A total of 43 TFs were available in GM12878 [71].

The peak files were mapped to the hg19 promoter regions. A peak overlapping at least one base with the promoter was considered to be a regulator of the promoter, the same as we mapped our LANA peaks. The transcripts with both LANA and ENCODE TFBS peaks in their promoters were identified. The distance between the center of the LANA peak and the ENCODE peak within the same promoter was calculated and plotted (e.g. CTCF and STAT1 in GM12878 vs. BCBL1). In the cases that multiple peaks exist in a promoter, the smallest distance was retained.

#### Motif analysis and clustering

We used the “peak motifs” tools from Regulatory Sequence Analysis Tools (RSAT, http://rsat.ulb.ac.be/rsat/) [69], [70], which is a web-based pipeline for discovering motifs from ChIP-Seq peak sequences. The fasta sequences of the identified peaks were retrieved using the BEDtools function fastaFromBed. To cluster LANA-enriched and epigenetic histone modifications, all LANA-enriched peaks were analyzed by seqMINER_1.3.3 using default setting and a range of plus/minus 5 kbp around annotated TSS [94].

#### Transient transfection assays

Transient transfection assays were performed as previously described [20]. In brief 293 cells were co-transfected with Luciferase reporter plasmid and various amount of LANA expression plasmid. At 72 hours post transfection, cells were harvested and lysed to assay the luciferase units, which is normalized to the total protein levels.

#### Quantitative PCR coupled with ChIP (ChIP-qPCR)

ChIP–qPCR was performed on an ABI Real-time PCR system using SYBR green (ABI) according to the manufacturer's instruction using the following program: Step 1: 95°C 20 sec; Step 2: 95°C 3 s, 60°C 30 sec, repeat 39 times; Step 3: melting curve from 60°C to 95°C. Results were analyzed using ABI StepOne Software. Enrichment of each region bound by histone mark or Pol II was calculated relative to its input standard curve. Values are expressed as percent of total chromatin for each bound region. Primer pair sequence for LANA promoter region is 5′-TATAGCATTTCAAAGATAAGGGTGC-3′ and 5′- CACCAATCAGAAAGTAGCTTGATAT-3′. Primer pair for vIL6 promoter is 5′- ACGTTCTTGAAAAACCCTCTCTTG -3′ and 5′- GCACAGCAAATTGACAAGGT -3′. K7 promoter primers are 5′-CGTGTTGGCGGGTTTGACCA-3′ and 5′- ACCCCCCGCCCAGTTATTCA-3′. ORF19 primers are 5′-GGCGAAAAAGTCAGCGGTGGT-3′ and 5′-CGGCGCGTCTTCCCTAAAGA-3′. Primers for RTA promoter are 5′-CCACTAAATACCAGGCAGCTA C-3′ and 5′-CAACAGACTACCCCTTGCG-3′.

#### EMSA

DNA duplexes were created by annealing the oligonucleotide pairs listed in Supplementary Table S6, end-labeled with [γ-^32^P]-ATP, and incubated with purified LANA-C protein as described previously [20]. Complexes were visualized following electrophoresis in native 4% polyacrylamide gels.

#### Western blot

Western blot analysis was performed as previously described [32]. 1×10^5^ BJAB, BCBL-1, TIVE, or TIVE-LTC cells were harvested and washed once with PBS. Cells were lyzed with 100 ul RIPA buffer. 20 ul of the cells lysate was separated in 8% SDS-PAGE gels and transferred to PVDF membrane. Membranes were blocked for 2 hours in T-TBS buffer containing 5% fat-free milk. Primary antibodies against specific proteins were diluted according to manufacturers' instruction and hybridized with membrane at 4°C overnight. After washing, 1∶5000 diluted corresponding secondary antibodies conjugated with peroxidase were incubated with the membrane for 1 hour at room temperature. After final washing, the blots were developed with ECL substrates (Millipore) and exposed to films.

#### Immunoprecipitation

IP experiments were performed as described before with minor modifications [32], [95]. 1×10^7^ cells were harvested and resuspended in 1 ml hypotonic buffer (10 mM HEPES pH 7.5, 1.5 mM MgCl_2_, and 10 mM KCl with protease inhibitor cocktail). Nuclei were spun down and resuspended in hypertonic buffer (20 mM HEPES pH 7.5, 20% glycerol, 500 mM NaCl, 1.5 mM MgCl_2_, 0.2 mM EDTA, 0.1% Triton X-100 and 1 mM DTT with protease inhibitors). Cells were lyzed at 4°C for 15 minutes with rotation. Dilution buffer which had the same ingredients as hypertonic buffer except for NaCl was added to make the final NaCl concentration 150 mM. Cell lysate was centrifuged for 10 minutes and pre-cleared with 50 µl protein A/G beads. A small portion of the lysate was saved as input. Cell lysate was incubated with 10 µg antibody at 4°C overnight with rotation. 100 µl protein A/G beads were added and incubated for 2 hours. The beads were collected by pulse centrifugation and washed 5 times with 1 ml ice-cold PBS with 0.1% Tween-20. Proteins were eluted with 1× Laemmli buffer and subjected to Western blot analysis.

## Supporting Information

Figure S1 **Bland-Altman Plot to investigate the agreement between two replicates of the ChIP-seq experiments.** RPKM was calculated for each sample after Bowtie alignment. The Y axis is the difference between two biological replicates and the X axis is the average between biological replicates. Green lines are the average of all differences +/−1.96× (standard deviation of the differences), indicating 95% confidence interval. The red line is drawn at zero.
(EPS)

Figure S2 **Distribution of H3K4me3, H3K27me3, Pol II, and LANA within the latency-associated region.** Detailed wiggle plots generated with UCSC genome browser illustrating nearly identical occupancy between latently infected BCBL-1 cells (A) and TIVE-LTC cells (B). Note distinct LANA, H3K4me3, and polII peaks within LANA ORF in TIVE-LTC cells (B).
(EPS)

Figure S3 **LANA binding within the terminal repeat and ORF50 regions.** Wiggle plots visualizing LANA occupancy on the viral TR and ORF50/RTA region. (A) Identical peak recovery from BCBL-1 and TIVE-LTC cells for binding to the LBS1/2 binding site within TR. Note very high recovery of LBS1/2 from BCBL-1 cells (y-axis). (B) Identical peak recovery within exonic sequences of RTA gene from both cell types. No LANA occupancy upstream of the RTA TSS was observed.
(EPS)

Figure S4 **Examples for cell type-specific and common LANA peaks within host promoters.** Wiggle plots generated with UCSC genome browser on selected LANA CHIP-seq-enriched promoters demonstrating cell type-specific (A–C) and (E–H) and common (D) occupancy between BCBL-1 and TIVE-LTC cells.
(EPS)

Figure S5 **Flow diagram of LANA peak identification and annotation.** Details about algorithms and programs used at each step are described in the Material and Methods Data analysis section.
(PPTX)

Table S1 **Comparison of KSHV nts sequences in Accession number NC_009333.1 and U75698.**
(DOCX)

Table S2 **Potential cellular targets regulated by LANA in BCBL-1 cells.**
(XLSX)

Table S3 **Potential cellular targets regulated by LANA in TIVE-LTC cells.**
(XLSX)

Table S4 **GO analysis of LANA targets in BCBL-1 cells.**
(XLSX)

Table S5 **GO analysis of LANA targets in TIVE-LTC cells.**
(XLSX)

Table S6 **Oligonucleotides used in EMSA assays.**
(XLSX)
