# Supplementary material for: LANA Binds to Multiple Active Viral and Cellular Promoters and Associates with the H3K4Methyltransferase hSET1 Complex
Source: PLoS Pathog. 2014 Jul 17;10(7):e1004240. doi: 10.1371/journal.ppat.1004240 (PMC4102568; doi:10.1371/journal.ppat.1004240)
Supplement: Table S1 — Comparison of KSHV nts sequences in Accession number NC_009333.1 and U75698. (DOCX) [file ppat.1004240.s006.docx]

**Supplemental Table 1: Comparison of KSHV nucleotide sequences in Accession # NC_009333.1 and U75698**

|  | U75698 | NC_009333 |
| --- | --- | --- |
| ORF73 major transcription start site | 127,880 | 128,029 |
| ORF73 translation start site | 127,297 | 127,447 |
| CTCF 1^st^ | 127,365 -127,391 | 127,514 – 127,540 |
| CTCF 2^nd^ | 127,425 - 127,458 | 127.574 – 127,607 |
| CTCF 3^rd^ | 127,487 – 127,544 | 127,636 – 127,693 |
| LANA binding site at LANAp | 128,051 - 128,072 | 128,200 – 128,220 |
